# Initial nipple damages in breastfeeding women: analysis of photographic images and clinical associations

**DOI:** 10.1590/0034-7167-2022-0773

**Published:** 2023-12-08

**Authors:** Bárbara Tideman Sartorio Camargo, Adriana Sañudo, Denise Miyuki Kusahara, Kelly Pereira Coca

**Affiliations:** I Universidade Federal de São Paulo. São Paulo, São Paulo, Brazil

**Keywords:** Nipples, Pain, Wounds and Injuries, Breast Feeding, Clinical Decision-Making, Pezones, Dolor, Heridas y Lesiones, Lactancia Materna, Toma de Decisiones Clínicas, Mamilos, Dor, Ferimentos e Lesões, Aleitamento Materno, Tomada de Decisão Clínica

## Abstract

**Objective:**

to analyze the initial nipple damage degree by breastfeeding practice and to associate findings with clinical manifestations of breastfeeding women.

**Methods:**

a retrospective, cross-sectional study with primary data and photographic images database from two randomized clinical trials. Photographic images were analyzed by two independent evaluators using the Nipple Trauma Score. For analysis, the chi-square, Mann-Whitney tests and Kappa coefficient were applied.

**Results:**

115 breastfeeding women and their respective 186 photographic images were analyzed. The degree of agreement of evaluators using the Nipple Trauma Score was 93.6%. The nipple pain score during breastfeeding was moderate and compromised more than 25% of the nipple surface area.

**Conclusions:**

assistance to breastfeeding women should prioritize nipple pain intensity instead of the nipple damage size.

## RESUMO

**Objetivo:**

analisar o grau de comprometimento tecidual das lesões mamilares precoces decorrentes da amamentação e associar achados com as manifestações clínicas de mulheres em amamentação.

**Métodos:**

estudo retrospectivo, transversal, envolvendo o uso de dados primários e de banco de imagens fotográficas provenientes de dois ensaios clínicos randomizados. Imagens fotográficas foram analisadas por duas avaliadoras independentes a partir do instrumento *Nipple Trauma Score*. Para análise, aplicou-se os testes Qui-Quadrado, Mann-Whitney e coeficiente Kappa.

**Resultados:**

foram analisadas 115 lactantes e respectivas 186 imagens fotográficas. O grau de concordância das avaliadoras pelo instrumento *Nipple Trauma Score* foi de 93,6%. O nível de dor mamilar encontrado durante as mamadas é moderado e há presença de lesões mamilares com mais de 25% de área da superfície do mamilo comprometida.

**Conclusões:**

a assistência a mulheres que amamentam deve priorizar o nível de dor apresentado em detrimento do tamanho da lesão mamilar.

## RESUMEN

**Objetivo:**

analizar el grado de afectación tisular en las lesiones tempranas del pezón resultantes de la lactancia materna y asociar los hallazgos con las manifestaciones clínicas de las mujeres que amamantan.

**Métodos:**

estudio retrospectivo, transversal, con datos primarios y banco de imágenes fotográficas de dos ensayos clínicos aleatorizados. Las imágenes fotográficas fueron analizadas por dos evaluadores independientes, utilizando el Nipple Trauma Score. Para el análisis se aplicaron las pruebas de Chi-Cuadrado, Mann-Whitney y coeficiente Kappa.

**Resultados:**

se analizaron 115 mujeres lactantes y sus respectivas 186 imágenes fotográficas. El grado de acuerdo de los evaluadores utilizando el Nipple Trauma Score fue del 93,6%. El nivel de dolor en el pezón durante la lactancia es moderado y existe presencia de lesiones del pezón con más del 25% de la superficie del pezón comprometida.

**Conclusiones:**

la asistencia a la mujer lactante debe priorizar el nivel de dolor que presenta en detrimento del tamaño de la lesión del pezón.

## INTRODUCTION

Nipple damage is a common cause for the early cessation of exclusive breastfeeding (EBF), mainly due to the intense and limiting nipple pain related to poor latch-on during breastfeeding^(1-2)^. The presence of nipple damage is more frequent in the first postpartum week and affects approximately 29 to 76% of women who breastfeed^(3)^.

Early cessation suppresses the mothers and their infant from receiving the benefits that involve breastfeeding (BF) practice^(4)^, that is exclusively recommended in the first 6 months of life and prolonged for two years or more with healthy complementary feeding^(5)^. Cessation of BF can result in an increased rate of gastrointestinal and respiratory infections in children, in addition to contributing to poor nutritional status, high infant morbidity and mortality, especially in developing countries^(6)^.

According to data from the Brazilian National Survey on Child Nutrition (ENANI-2019)^(7)^, currently, the national prevalence of EBF among children younger than 6 months is 45.8%, a rate below the 70% recommended in the targets established by the World Health Organization (WHO), Sustainable Development Goals (SDGs) for 2030^(8)^.

The care of women with nipple damage during BF is an old challenge and, despite the extensive knowledge of its cause and the understanding of the importance of preventive guidelines, such as correct positioning and latch-on during BF, it is not always possible to prevent its occurrence^(9)^. The main factors that contribute to nipple damage appearance are those related to poor latch-on and positioning, and include not wide mouth (opening angle less than 140º), lips turned inward, symmetrical latch-on, chin far from the breast, body of the child misaligned and distant from the mother’s body, woman without support and with her body on top of the child^(10-11)^.

In addition, other factors refer to woman and child anatomy and breast care, categorized into: external influences (nipple size and shape, infant presence and length of ankyloglossia and use of pumps); modulation via central nervous system (maternal social and emotional conditions, past history and training); and local stimulus (nipple damage characteristics and healing factors)^(12)^.

As for nipple damage resolution, there is no consensus regarding the best treatment method for tissue repair and nipple pain relief^(13)^, in addition to identifying and correcting the cause^(14)^. A systematic review, which analyzed the treatment methods described in the literature, showed that there is no evidence enough to recommend any treatment for nipple pain, and enhances the importance of preventive guidelines for women to continue EBF^(13)^. The use of topical or oral treatments (fungal and bacterial infections) and non-pharmacological treatments (lanolin, photobiomodulation, hydration with expressed breast milk, nipple pads) were mentioned^(14-15)^. The results suggest the importance of prevention and proper management, including breastfeeding positioning and attachment support^(14-15)^.

Complaint of nipple pain, often assessed using the Visual Analog and Numerical Pain Scale^(15)^, may vary among women with or without damaged nipples and the postpartum period, being identified with higher scores in women with damaged nipple^(15-16)^ and with a reduction to mild scores after about 7 to 10 days postpartum, regardless of the treatment used^(13)^.

Nipple damages related to the BF phase can occur at different times, being more frequent in the first postpartum week due to the beginning and establishment of infant at the breast^(11)^, called initial nipple damages. Solving the problem of its initiation promotes its repair, regardless of the proposed treatment. However, complaints of nipple damages are also observed in more advanced postpartum periods, commonly related to fungal infections in the nipple-areolar complex^(17)^.

Systematic assessment contributes to the target on the resolution and opportune treatments, adequate to the process of tissue healing and pain perception, which confer the necessary celerity and allow the linear maintenance of BF. Thus, the identification of the types of nipple damages and the moment of their occurrence can contribute to differentiation of the causal factor and the proposed treatment.

Furthermore, nipple damage assessment is also related to management. Among the assessment resources and methods, the performance of a detailed clinical examination stands out, including the use of measuring instruments (scales, indices or scores) to measure the damages, use tools for better visualization (magnifying glasses, direct light) and recording by photographic images for evolutionary analysis of nipple damages^(18)^.

In this context, in nipple damage assessment, both the tissue damage degree and its morphology classification can contribute to a more specific treatment. It is known that nipple damages can reach the epidermis and/or dermis, and involve different skin structures^(18)^. In a recent study, nipple damages were classified according to the interruption of the cutaneous barrier in the nipple-areolar complex, in order to standardize terminologies and interpretations of their characteristics^(18)^.

This assessment is still being explored in the literature and its applicability in clinical practice, therefore, little used by health professionals, who still call nipple damages as fissures in a generalized way^(18)^. In this regard, in-depth studies on this subject become increasingly necessary so that appropriate treatments can be indicated.

## OBJECTIVE

To analyze initial nipple damage degree resulting from BF and to associated findings with the clinical manifestations of BF women.

## METHODS

### Ethical aspects

The study complied with Resolution 466/2012 of the Brazilian National Health Council, and data collection was carried out after approval by the Institutional Review Board of the *Universidade Federal de São Paulo*. The Informed Consent Form was obtained from all women involved in the study in writing prior to data collection.

### Study design, period and place

This is a retrospective, cross-sectional study with secondary analysis, which adopted the STrengthening the Reporting of OBservational studies in Epidemiology (STROBE) as a framework, involving the use of primary data and a database of photographic images, from two randomized clinical trials, conducted by researchers from the current study and developed with women with nipple damage in the initial lactation phase^(19-20)^. At the time, the authors received permission to obtain images of participants’ breasts, as approved by the Institutional Review Board^(19-20)^.

### Sample: inclusion and exclusion criteria

The sample consisted of 145 women and 252 photographic images of nipple damages, defined as skin barrier disruption^(18)^ and located at the nipple tip. The sampling technique used was non-probabilistic for convenience, where all photographic images obtained in both studies were analyzed^(19-20)^.

Participants were recruited for the primary studies (clinical trials) based on the inclusion criteria: women with a singleton; who had a birth weight equal to or greater than 2,500 grams; and EBF directly on the breast. Women with malformed nipples, mastitis or malignant disease were not included. As for secondary analysis, the exclusion of women whose images interfered with sharpness was added to the eligibility criteria, due to the environment’s natural lighting at the time of the photographic record, due to the difficulty in assessing the images.

### Variables and measurement methods

Nipple damages were analyzed from photographic records obtained during the execution of clinical trials prior to this secondary analysis. In clinical trials, photographic records were standardized in macro mode, under natural light, vertical orientation/portrait at 5 centimeters from the nipple-areolar complex, with the woman in supine position and leaning against the wall, using a SONY Cybershot DSC-W330 digital camera^(19-20)^.

The size refers to nipple damage length, measured using an acrylic ruler and presented in millimeters (mm). In the presence of multiple nipple damages, the one with the greatest extension was considered. Nipple pain score during BF was verified using the Visual Analog Scale (VAS, 0-10), in which, at the time of clinical trials, women reported pain intensity during the feeding in the affected breast (0= no pain and 10= worst pain imaginable)^(15,21)^. Pain was classified as absent (0 points), mild (1-3 points), moderate (4-6 points) and severe (7-10 points)^(15,21)^.

Regarding nipple damage tissue impairment degree, the Nipple Trauma Score (NTS)^(22)^ was used, which was translated into Portuguese via back-translation by the researchers (Chart 1). NTS characterizes nipple damage based on tissue injury depth and extent. The NTS ranges from 0 to 5, with 0 meaning no macroscopically visible changes in the skin and 5 a partial-thickness damage of more than 25% of the nipple surface, with or without scab formation. Nipple damage degree according to NTS was categorized into scores 2 and 3 for the application of hypothesis tests, with 2 being considered nipple damages with superficial damage with or without scab formation on less than 25% of the nipple surface (scores between 0-2 included) and 3 with superficial damage with or without scab formation on more than 25% of the nipple surface (includes scores between 3-5).

**Chart 1:** Nipple Trauma Score^(22)^: version translated by the researchers via back-translation. São Paulo, São Paulo, Brazil, 2023

Nipple Trauma Score (NTS)	
**Score**	**Characteristics**
0	*Não há mudança visível na pele do mamilo*
1	*Eritema ou edema ou ambos*
2	*Dano superficial com ou sem crosta formada em menos de 25% da superfície do mamilo*
3	*Dano superficial com ou sem crosta formada em mais de 25% da superfície do mamilo*
4	*Lesão com espessura parcial com ou sem crosta formada em menos de 25% da superfície do mamilo*
5	*Lesão com espessura parcial com ou sem crosta formada em mais de 25% da superfície do mamilo*

*Original source: Abou-Dakn M, Fluhr JW, Gensch M, Wöckel A. Positive effect of HPA lanolin versus expressed breastmilk on painful and damaged nipples during lactation. Skin Pharmacol Physiol. 2011;24(1):27-35. https://doi.org/10.1159/000318228*

Variables were classified, for analysis, into independent and dependent variables. The chosen independent variables were age (numerical, in complete years), parity (nominal, primiparous and multiparous), postpartum day of inclusion of women in the study (nominal, being 1^st^ day = less than 24 hours after birth and 2^nd^ day= between 24 and 48 hours after birth), breast condition (nominal, soft, turgid and/or engorged) and nipple damage side (nominal, one nipple or both nipples).

Associated dependent variables were damage size (numerical, in millimeters), nipple pain score during BF (ordinal, absent (0)/mild (1-3)/moderate (4-6)/severe (7-10) and degree of damaged nipples (nominal, NTS 2 and NTS 3 categorization).

### Study protocol

Data collection was carried out during the rooming-in unit period, with photographic images obtained in the first two postpartum days, between 2011 and 2017, in São Paulo, Brazil^(19-20)^. Photographic image analysis was carried out between October 2021 and September 2022. Variables were extracted from the databases of the respective studies by the researchers, after performing data grouping and universal coding via Excel^®^. For tissue damage degree analysis, photographic images in a hard-copy version were used, randomly ordered and identified specifically for this study.

A developed instrument constructed specifically for this study was used, in which evaluators recorded the assessment parameters of nipple damages when evaluating the images.

**Figure 1:**
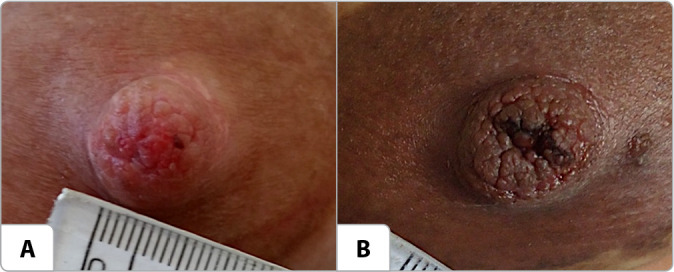
Photographic images of nipple damages included. São Paulo, São Paulo, Brazil, 2023

Photographic images were analyzed by two independent evaluators, International Board Certified Lactation Consultant (IBCLC) nurse-midwives, English speakers and minimum experience of 10 years in BF clinical management. Then, the photographic images assessments were compared, and divergences were discussed among evaluators, and in case of disagreement a third evaluator was called (Figure 1).

### Analysis of results, and statistics

Descriptive analysis included mean or median, standard deviation or interquartile range for numeric variables. The Shapiro-Wilk test was used to assess the normal distribution of data, considering p<0.05, which identified a non-parametric sample.

Categorical variables were measured by simple frequency and percentage. To verify the association between dependent and independent variables, the chi-square and Mann-Whitney tests were used.

The Kappa coefficient was used to measure the researchers’ degree of agreement regarding nipple damage, considering the following values: <0.00, insignificant agreement; 0.00-0.20, poor agreement; 0.21-0.40, fair agreement; 0.41-0.60, moderate agreement; 0.61-0.80, strong agreement; and 0.81-1.00, almost perfect agreement^(23)^. Data were tabulated using Excel^®^ (Microsoft, USA) and inferential analysis using the STATA 14 statistical software (Stata#Corp, USA). The level of statistical significance was set at 0.05.

**Figure 2:**
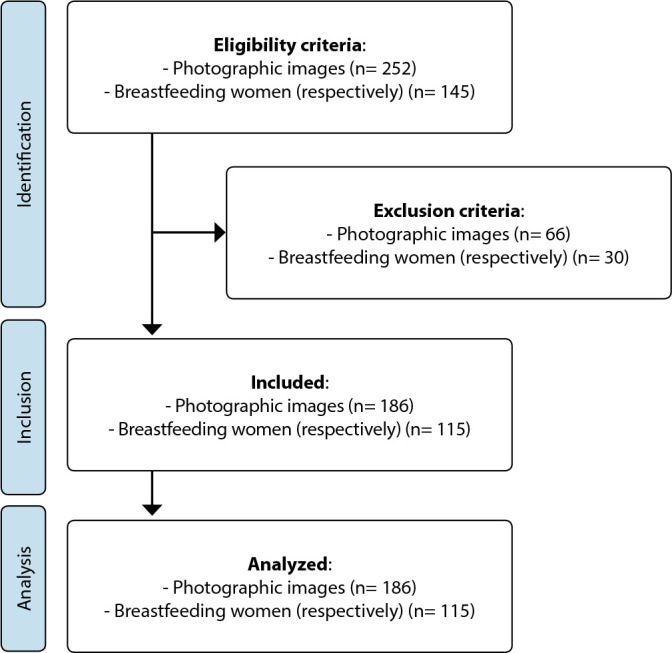
STrengthening the Reporting of OBservational studies in Epidemiology flowchart. São Paulo, São Paulo, Brazil, 2023

Data collection according to the STROBE framework is shown below (Figure 2).

## RESULTS

Data from 145 BF women and 252 photographic images of nipple damage resulting from BF were analyzed. Of these, 66 images were excluded for not presenting nipple damage with macroscopic clarity, which corresponded to the exclusion of 30 women, composing the final sample: 115 BF women and their respective 186 photographic images.

BF women’s median age (P25-P75) was 26 years (21/31), the majority being primiparous (52.2%), with soft breasts (92.1%), nipple damage in both nipples (79.1%) and with nipple damage occurring on the 1^st^ day after birth (64%) (Table 1).

**Table 1:** Distribution of characterization data for women and nipple damages. São Paulo, São Paulo, Brazil, 2023

Variables	Total
Woman characteristics	
Age (years)	
Median (p25-p75)	26 (21-31)
Parity (n= 115) n (%)	
Primiparous	60 (52.2)
Multiparous	55 (47.8)
Condition of the breasts (n=115) n (%)	
Soft	106 (92.1)
Turgid/engorged	9 (7.8)
Nipple damage side (n=115) n (%)	
One nipple	24 (20.9)
Both nipples	91 (79.1)
Nipple damage characteristic	
Inclusion postpartum day (n=186) n (%)	
1^st^ postpartum day	119 (64)
2^nd^ postpartum day	67 (36)

In Table 2, data were related to nipple pain score presented by women and nipple damage size. With regard to nipple pain score (VAS) during BF, moderate pain was identified, with a median of 5 points in both breasts, while the median nipple damage size was 8 mm and 9 mm in the right and left breasts, respectively. Most women (82.8%) had a degree of superficial damage with or without scab formation on more than 25% of the nipple surface (Table 2).

**Table 2:** Nipple pain score presented by women and nipple damage size: descriptive analysis. São Paulo, São Paulo, Brazil, 2023

Dependent variables	P50	P25-P75
Nipple pain score (VAS^*^)		
Right breast (n= 90)	5	0-8
Left breast (n= 96)	5	1.5-8
Nipple damage size (mm)		
Right breast (n= 90)	8	5-10
Left breast (n= 96)	9	7-11

* VAS= Visual Analog Scale;

** NTS 2= score attributed to nipple damage degree comprised between scores 0-2;

*** NTS 3= score assigned to nipple damage degree comprised between scores 3-5.

It is noteworthy that the agreement degree among evaluators in the nipple damage degree classification by NTS was 93.6%. The Kappa coefficient value for internal reliability was considered almost perfect (k= 0.82).

The association between initial nipple damage size and evaluators’ classification regarding damage degree by NTS revealed to be statistically significant. The greater the median size of the nipple damage, the greater the tissue damage degree (p<0.001).

Regarding nipple damage degree and nipple pain score, it was observed that the median pain of women with a superficial damage with or without a scab formation in less than 25% of the nipple surface (NTS 2) was higher when compared to the median of pain reported by women with a superficial damage with or without a scab formation on more than 25% of the nipple surface (NTS 3), but without statistical significance.

No associations were observed between nipple pain score and the moment of occurrence of nipple damage with degree of nipple damage according to NTS.

Associations between dependent variables are shown in Table 3, below.

**Table 3:** Associations between dependent variables: nipple damage size, nipple pain score to breastfeed and inclusion postpartum day, São Paulo, São Paulo, Brazil, 2023

Variáveis dependentes	n	Median (P25-P75)	*p* value
Nipple damage size (mm)			**< 0.001^A^**
NTS 2^*^	32	5 (4-5)	
NTS 3^**^	154	9 (7-11)	
Nipple pain score (VAS^***^)			0.055^A^
NTS 2^*^	32	7 (4.5-8)	
NTS 3^**^	154	5 (0-7)	
Postpartum inclusion day (1^st^ or 2^nd^ day)			0.551^B^
NTS 2^*^	32	1 (1-2)	
NTS 3^**^	154	1 (1-2)	

* NTS 2= score assigned to nipple damage degree comprised between scores 0-2;

** NTS 3= score assigned to nipple damage degree comprised between scores 3-5;

*** VAS= Visual Analog Scale; A= Mann-Whitney test; B= chi-square test.

## DISCUSSION

The nipple pain score found in the first two postpartum days during feedings is moderate, and the compromised area of nipple damage is more than 25% of the nipple surface. Women’s pain score during BF and the moment of occurrence of nipple damage did not affect nipple damage degree.

This study presents a new theme in the literature by assessing the degree of nipple damage using NTS based on photographic images, comparing nipple damage size and nipple pain score associated with BF. The importance of exploring these approaches stands out in the implementation of differential clinical approaches that promote tissue regeneration of nipple damage and thus favor EBF duration.

There is a limit to classifying the nipple damage type, associated with the postpartum period of occurrence and nipple damage degree, which lead the professional to a different interpretation, generating conducts that are sometimes generalized and not based on scientific evidence.

Nipple damages can be classified according to their postpartum period, i.e., according to the period in which they appear. They can occur from infants’ first contact with the breast, with the main associated and determining cause being inadequate latch-on and positioning during BF^(13,24-27)^. Inexperience with BF attributed to both infants and BF women is considered relevant during this period, regardless of whether they have already breastfed previously, which may contribute to this difficulty^(9)^. These nipple damages can be defined as initial given their abrupt, acute onset process and relatively determined cause.

Initial nipple damage can occur in one or both nipples. In this study, the majority of nipple damages occurred on both nipples, a condition that translates into an even greater challenge for women to maintain breast supply.

Nipple pain score reported during BF by women was assessed individually between breasts and, in both the right and left breasts, moderate intensity was reported, corresponding to 4 and 5, respectively. This data is consistent with the available literature^(15)^ that, in a systematic literature review, found a weighted mean pain intensity of 6.2 on VAS during BF in the first week postpartum. Other comparative studies between treatments for early nipple damage also revealed moderate pain intensity in women with nipple damage who were BF during the same period^(28-31)^.

Nipple pain score during BF may have repercussions on other situations experienced by BF women and lead to interruption of BF, such as a reduction in milk ejection reflex as a result of reduction or limitation of supply from the damaged breast, which has an impact on the suckling pressure, which may contribute to nipple damage worsening and persistence, in addition to predisposing to occurrence of complications, such as local fungal and bacterial infections, disseminated to the breast^(22,32-34)^.

The mean size of nipple damages in this study were 8.3 mm in the right breast and 8.8 mm in the left breast, with variations between 2 mm and 23 mm. Establishing nipple damage size in order to assess tissue healing based on the proposed treatments and guidelines represents a challenge for practice. It was considered that the parameters usually used to characterize this evolution, such as area of damage, amount of exudate and appearance of pressure ulcer (Pressure Ulcer Scale for Healing (PUSH))^(35)^, are sometimes infeasible in these nipple damages, since they have irregular edges, different diameters, depth that is difficult to measure and appearance and amount of exudate influenced by the humidity of infants’ oral mucosa when BF.

Efforts to standardize nipple damage assessment considering their peculiarities have been explored. Recently published, the *Instrumento de Classificação das Lesões Mamilo-Areolares* (ILMA)^(36)^ brings in its proposal the unification of the nomenclature of nipple damages under a dermatological approach, differentiating damages without skin barrier interruption (erythema, ecchymosis, edema and vesicle) and with skin barrier interruption (fissure, erosion and crust). Another study, also with the aim of establishing terminologies based on consensus among experts, proposes the instrument “Seven Signs of Nipple Trauma Associated with Breastfeeding” (Erythema, Swelling, Scabbing, Blistering, Fissure, Purpura e Peeling), which relies on images to guide the assessment^(37-38)^. Differentiating nipple damages is essential to direct treatments and conduct, in order to obtain satisfactory results.

The tissue impairment degree measured using NTS revealed scores attributed to all scores, demonstrating variations related to the depth and extent of nipple damages observed between the first 48 hours postpartum.

Identifying nipple damage degree is fundamental for establishing clinical management. NTS used to assess nipple damages proved to be valid, accurate and easy to apply, with potential for use in practice, demonstrated by the high agreement degree among evaluators in this study and in a study with a similar theme^(22)^.

In this study, nipple damages classified as NTS 2 had a higher nipple pain score than those classified as NTS 3, revealing that pain intensity should be valued, regardless of nipple damage size. Considering the findings, it is recommended that the moment of onset of nipple damage be considered, i.e., initial, late or persistent, since there is variation between related causes.

The advancement of classification and associations with the various characteristics of the damage will bring great benefit to BF women with this condition, contributing to direction of care during the lactation phase. Health education for BF women focused on appropriate BF techniques performed in the postpartum period continues to be important in preventing nipple damage; however, assertive treatment according to damage specifications can reduce early cessation rates^(39)^.

### Study limitations

This is a secondary analysis study based on primary data and respective photographic images on the first and second day after birth. During this period, the nipple pain in initial nipple damage from infants’ first exposure to the breast may influence the occurrence of higher pain scores, while, on the second day, BF women may have familiarized with the breastfeeding practice, influencing the perception of nipple pain reduction. Deleting images was also a limitation. Setting the starting day of nipple damage instead of the postpartum day may provide better results.

### Contributions to nursing, health or public policies

The results of this paper contribute to health professionals’ clinical decision-making, with emphasis on nurses who assist BF women during infants’ first exposure to the breast. Broadening the view of the nipple pain during BFs to the detriment of the existence or not of a visible nipple damage can both prevent its occurrence and guide actions such as good latch and positioning and proposing appropriate treatment procedures. Additional studies that propose treatments according to the specified characteristics of nipple damages are necessary.

## CONCLUSIONS

Nipple pain score in BF women who have nipple damage is high on the first day postpartum, regardless of nipple size and damage degree. Professionals who assist postpartum women during infants’ first exposure to the breast must understand the need to frequently monitor these feedings, directing their attention to preventing nipple damage occurrence and worsening.

Expanding the clinical perspective beyond nipple damage size is also necessary. It is essential that the focus of care is on nipple pain score during BF, and not on nipple damage size and degree. Initial nipple damages resulting from BF can impact continuity of BF and mothers’ and children’s quality of life during this practice.
